# Tigecycline-Amikacin Combination Effectively Suppresses the Selection of Resistance in Clinical Isolates of KPC-Producing *Klebsiella pneumoniae*

**DOI:** 10.3389/fmicb.2016.01304

**Published:** 2016-08-19

**Authors:** Wentao Ni, Chuanqi Wei, Chufei Zhou, Jin Zhao, Beibei Liang, Junchang Cui, Rui Wang, Youning Liu

**Affiliations:** ^1^Department of Respiratory Diseases, Chinese People’s Liberation Army General HospitalBeijing, China; ^2^School of Pharmacy, Shenyang Pharmaceutical UniversityShenyang, China; ^3^Department of Clinical Pharmacology, Chinese People’s Liberation Army General HospitalBeijing, China

**Keywords:** MPC, MSW, tigecycline, colistin, amikacin, *Klebsiella pneumoniae*

## Abstract

By far, only tigecycline, colistin, and some aminoglycosides still show favorable *in vitro* activities against carbapenem-resistant *Enterobacteriaceae*. However, rapid emergence of resistance often occurs during long-term treatment in clinic, challenging these last resort antimicrobials. In this study, we measured mutant prevention concentration (MPC) and mutant selection window (MSW) of tigecycline, colistin and amikacin alone and in combination for clinical isolates of KPC-producing *K. pneumoniae*, and characterized the resistant mutants recovered. The MPC_90_ of 30 tested isolates for tigecycline, colistin, and amikacin were 16, >128, and 128 mg/L, respectively. The average MSW of tigecycline-amikacin, tigecycline-colistin, and amikacin-colistin combinations for four representative strains were 11.99, 200.13, and 372.38, respectively. A strong correlation was found between the MSW*_combination_* and the product of MSW of each single drug. Combinations of 1 minimal inhibitory concentration (MIC) multiple tigecycline and 1 MIC multiple amikacin could result in 1000- to 10000-fold reduction in mutational frequency relative to their individual mutational frequencies, and combinations of 1 MIC multiple amikacin and 1.5–2 MIC multiple tigecycline could successfully restrict the recovery of resistant mutants on agar plates. However, 2 MIC multiple colistin in combination with 2 MIC multiple tigecycline or amikacin merely resulted in approximately 10-fold decrease in the mutational frequency. In conclusion, this study showed tigecycline-amikacin combination could effectively suppress the selection of resistance at low concentrations compared with the colistin-tigecycline and colistin-amikacin combinations, suggesting that this combination may be useful in clinical therapy.

## Introduction

The worldwide emergence and dissemination of carbapenem-resistant *Enterobacteriaceae* (CRE), particularly *Klebsiella pneumoniae* producing *Klebsiella pneumoniae* carbapenemase (KPC), represents a serious threat to public health (Munoz-Price et al., 2013). Besides carbapenems, these superbugs often carry genes that confer high levels of resistance to many other broad-spectrum antibiotics (Hirsch and Tam, 2010). By far, only a few agents, such as tigecycline, colistin and some aminoglycosides show favorable *in vitro* activities against CRE (Hirsch and Tam, 2010). However, due to the wide use, resistance has challenged these last-resort treatment options (Capone et al., 2013; Monaco et al., 2014; Giske, 2015).

Since the pace of developing new effective antibiotics is quite slow, suppressing further resistance emergence of current available agents is of the great significance. Selective enrichment and amplification of resistant mutant isolates most likely occurs when antimicrobial concentrations fall in a specific range called the mutant selection window (MSW). The lower boundary of the MSW is approximate to the minimal inhibitory concentration (MIC), and the upper boundary is the mutant prevention concentration (MPC), which could prevent the emergence of all least susceptible, single-step resistant mutant subpopulations (Zhao and Drlica, 2008). The spontaneous single-drug resistant mutants usually arise at a frequency from 10^-6^ to 10^-8^, and initially susceptible bacteria theoretically may have a fairly low probability (<10^-10^) to develop mutations causing resistance to both drugs for surviving at concentrations above their respective MICs (Zhao and Drlica, 2001). Thus, simultaneous administration of two antibiotics with different modes of action (without occurrence of cross-resistance) may suppress the selection of resistant mutants (Zhao and Drlica, 2001). In addition, many studies have reported favorable clinical outcomes and lower mortality rates for patients treated with combination therapies (Falagas et al., 2014; Ni et al., 2015). Therefore, for improving the efficacy and preventing the emergence of further resistance, most clinicians recommend using combination therapies to treat CRE infections (Rafailidis and Falagas, 2014).

The aim of this study was to determine the *in vitro* effects of tigecycline, colistin and amikacin alone and in combination on recovery of resistant mutants of KPC-producing *K. pneumonia*, and to predict the ability of different antibiotic combinations on the prevention of resistance emergence during treatment in clinic.

## Materials and Methods

### Bacterial Strains and Antimicrobial Agents

Sixty-eight identified KPC-producing *K. pneumoniae* strains were isolated from different patients during the period June – December 2014 from the department of clinical microbiology in two tertiary hospitals in Beijing, China. *Escherichia coli* ATCC25922 was used as a reference strain. All strains were identified using the Vitek^®^ 2 Compact System (bioMérieux, Marcyl’Étoile, France).

Tigecycline and colistin standards were purchased from Sigma-Aldrich (St Louis, MO, USA). Amikacin standard was obtained from the National Institute for the Control of Pharmaceutical and Biological Products (NICPBP, Beijing, China). All the agents were prepared as fresh stock solutions in sterile distilled water on the day of use.

This study was performed in a standard Microbiological Lab in our hospital. We handle the clinical isolates according to the WHO Laboratory Biosafety Manual (WHO, 2004).

### Determination of the MICs

The MICs of tigecycline, colistin, and amikacin were determined by the agar dilution method according to CLSI (2014) guidelines. In brief, Mueller Hinton agar (Difco, Franklin Lakes, NJ, USA) plates containing a series of twofold concentration increments of each agent were prepared according to specifications. Then, approximate 10^4^–10^5^ CFU bacterial cells were inoculated by an autoclaved replicator and incubated at 35°C in ambient air for 20 h. The MIC was defined as the lowest drug concentration that inhibited the visible growth of colonies. All MIC determinations were conducted at least in triplicate on separate days. The MIC breakpoints of susceptibility for the three antibiotics were interpreted in accordance with the European Committee on Antimicrobial Susceptibility Testing standards (EUCAST): ≤1 mg/L for tigecycline, ≤2 mg/L for colistin, and ≤8 mg/L for amikacin (EUCAST, 2015).

### Enterobacterial Repetitive Intergenic Consensus-PCR (ERIC-PCR)

In Brief, each strain underwent Enterobacterial Repetitive Intergenic Consensus-PCR (ERIC-PCR) typing as described previously (Smith et al., 2007; Ying et al., 2015).The primer pair ERIC1 and ERIC2 (**Supplementary Table 1**) were used to amplify intervening fragments of ERIC in the genomic DNA. Each reaction contained 2.5 μl of 10x PCR buffer, 2 μl dNTP mixture, 0.125 μl Taq enzyme, 0.5 μl each primer (10 μmol/L) and 1 μl DNA template, added to a final volume of 25 μl with ddH_2_O. Thermal cycling was performed in an ABI 2700 thermal cycler (Applied Biosystems, Carlsbad, CA, USA): initial incubation at 94°C for 7 min; 30 cycles of 30 s at 94°C, 1 min at 52°C, and 8 min at 65°C; and a final incubation at 65°C for 10 min. The amplified products were resolved in 1.2% agarose gel electrophoresis stained with ethidium bromide. The data were analyzed with the software package BioNumerics 5.1 (Applied Maths, Austin, TX, USA) as previously described (Smith et al., 2007).

### Determination of the MPCs and Frequency of Resistant Mutants

The MPCs of drugs alone and in combinations were determined by methods described previously (Zhanel et al., 2006; Ni et al., 2013). In brief, bacterial cells were grown overnight in fresh Mueller-Hinton broth (MHB) with vigorous shaking at 35°C in ambient air. Then the overnight cultures were diluted by 10-fold with MHB, and incubated at 35°C for 6 h. The growth was centrifuged (4000 × *g* for 5 min) to yield a high-density culture containing cells of ∼3 × 10^10^ CFU/ml. Fifty microliters of samples (approximate 1.5 × 10^9^ cells) were inoculated onto Mueller-Hinton agar plates (diameter, 90 mm) containing different concentrations of tigecycline, colistin and amikacin alone or in combination. For a given drug concentration, seven plates were used to ensure that the total number of cells tested was at least 10^10^. For counting the number of resistant colonies arising at a given concentration, another eight plates was inoculated with different number of bacterial cells (10^3^, 10^5^, 10^7^, and 10^8^). At the same time, the cell density of the culture was determined retrospectively by applying serial dilutions to drug-free agar plates. All plates were incubated at 35°C in ambient air for 3 days. The MPC was defined as the concentration that blocked growth when at least 10^10^ cells were applied to agar plates. The number of colonies arising in each plate of a given concentration was counted (if it was countable, typically,<500 CFU per plate) and the frequency of resistant mutants was defined as the total number of colonies in all plates with countable colonies divided by the total number of cells plated on these specific plates.

### Measurement of the MSWs of Single Drugs and Combinations

**FIGURE 1:**
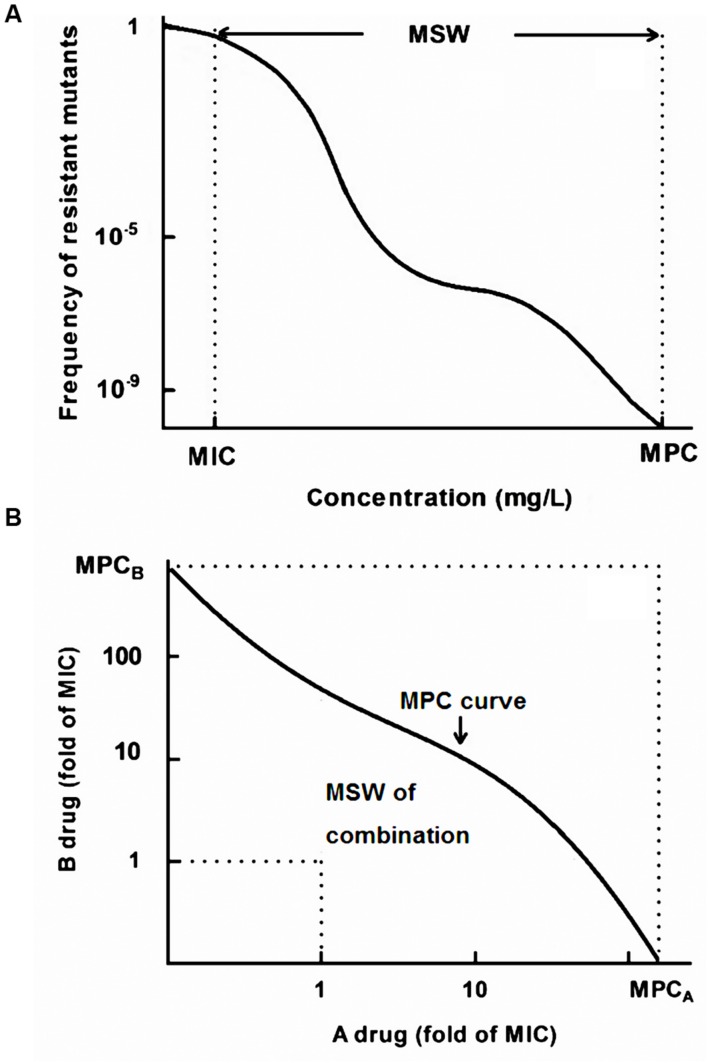
**A theoretical model of the mutant selection window (MSW).** **(A)** MSW of a single drug ranging from the MIC to the MPC; **(B)** The MSW notion in drug combinations extends from the MPC curve to the MIC line (dashed line).

As mentioned above, the MSW of a single drug is a concentration range which approximately extends from the MIC to MPC, denoted by double-headed arrows in **Figure 1A**. The width of MSW can be expressed as a ratio (MPC/MIC), which is also termed as selection index (SI). As displayed in **Figure 1B**, no resistant mutants can recover above the MPC curve, and the MIC lines are approximate the lower boundary of the MSW in combination therapy (Zhao and Drlica, 2002). Therefore, the MSW of drug combinations (MSW*_combination_*) can be measured as the area that extends from the MPC curve to the MIC lines of each drug (Zhao and Drlica, 2002).

### Characterization of Resistant Mutants

For examining whether the arising colonies of each plate at a given concentration contain stable resistant mutants, two colonies of each plate were passaged five times on drug-free agars, and then the MICs were determined by the agar dilution methods. Real-time quantitative reverse transcription PCR (qRT-PCR) was used to detect the expression levels of the efflux pump gene *acrB* and the global transcriptional regulator *ramA* in tigecycline-resistant mutants, the expression level of *pmrK* that belongs to the *pmrHFIJKLM* operon in colistin-resistant mutants and the efflux pump gene *acrD* in amikacin-resistant mutants. The qRT-PCR was performed as previously described (He et al., 2015). Briefly, overnight bacterial cultures were diluted with MHB broth, and then were grown at 35°C with shaking of 200 rpm. Cell pellets were harvested at OD_600_ = 0.6. The Purelink RNA Mini Kit (Ambion, Carlsbad, CA, USA) was used for extracting ribonucleic acid (RNA). The yield and quality of RNA were determined by a Nanodrop 2000C (Thermo, USA). Then the total RNA was reverse transcribed into cDNA using the PrimeScript RT Reagent kit (Tiangen, Beijing, China). Real-time qRT-PCR was performed using a LightCycler 480 II (Roche, Germany) with 40 cycles of 15 s at 95°C, 20 s at 52°C, and 30 s at 72°C, and SYBR Premix Ex Taq (TaKaRa, Dalian, China) was used to quantify the expression of the target gene. Expression of each gene was normalized to that of a housekeeping gene (*rrsE*). The parent strain was used as the reference strain. Besides, the *mgrB* gene of the colistin-resistant mutants and the aminoglycoside-modifying enzymes genes including *aac (3)-I*, *aac (3)-II*, *aac (3)-III*, *aac (6)-Ib*, *aac (6)-II*, *ant (2″)-I*, *ant (3″)-I*, and 16S rRNA methylase genes (*armA*, *rmtA*, *rmtB*, *rmtC*, *rmtD*, *npmA*) of amikacin-resistant mutants was amplified by PCR, and then the *mgrB* was sequenced by the Applied Biosystems 3730 sequence analyser (Applied Biosystems Inc., USA). In brief, the whole-cell DNA was extracted using the TIANcombi DNA Lyse & Det PCR Kit (Tiangen, Beijing, China) according to the manufacturer’s instructions. Each reaction contained 2.5 μl of 10x PCR buffer, 2 μl dNTP mixture, 0.3 μl Taq enzyme (5 U/μl; Tiangen, Beijing, China), 1 μl each primer (10 μmol/L) and 1 μl DNA template and 17.2 μl ddH_2_O. The PCR method consisted of initial incubation at 94°C for 2 min; 35 cycles of 20 s at 94°C, 20 s at 55°C, and 30 s at 72°C; and a final incubation at 72°C for 10 min. The primers used in this study were shown in **Supplementary Table 1**.

### Statistical Analysis

MSW*_combination_* of different drug pairs were compared and their differences were determined by One-way ANOVA and independent-sample *t*-test. We speculated the MSW*_combination_* might be associated with the MSW of each single drug. Therefore, the relationship between the MSWs of single drugs and the MSW*_combination_* was evaluated by Pearson’s correlation analysis. All analyses were performed with the software of SPSS 20.0. A *P*-value < 0.05 was considered statistically significant.

## Results

### MPCs and MSWs of Single Drugs

**FIGURE 2:**
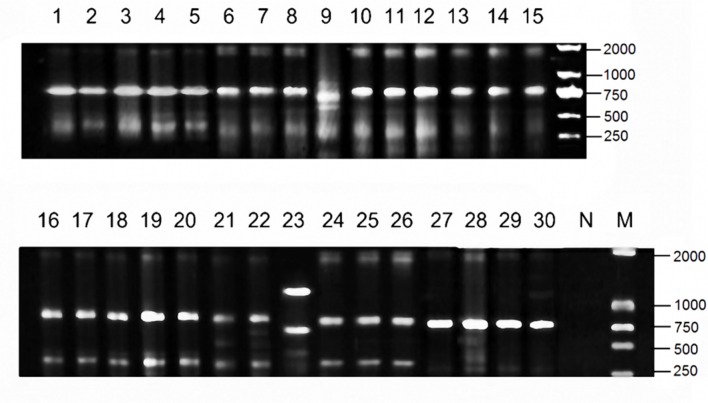
**Electrophoretogram of PCR product in Enterobacterial repetitive intergenic consensus sequence (ERIC) profiles of 30 KPC-producing *Klebsiella pneumoniae* clinical isolates investigated in this study**.

**FIGURE 3:**
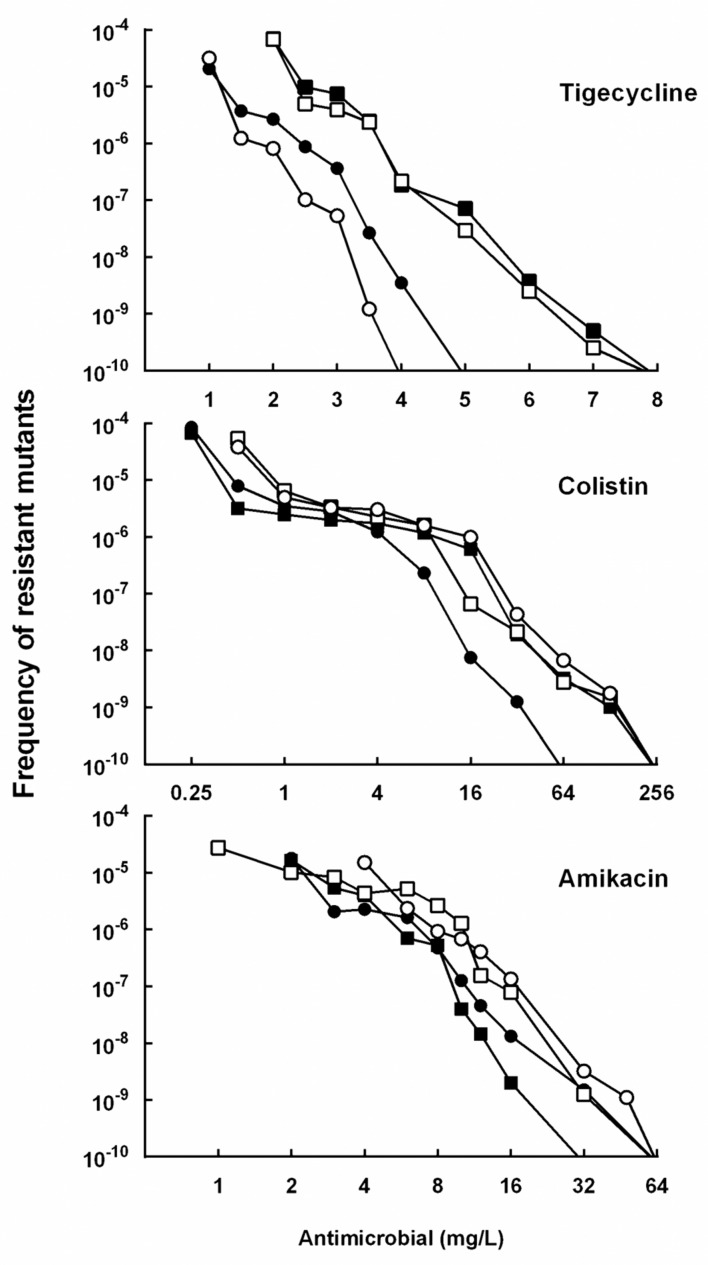
**Effect of antimicrobial concentration on recovery of mutants in *K. pneumoniae* strain Kp3 (filled circles), Kp18 (filled squares), Kp8 (open circles), and Kp23 (open squares) were applied to agar plates containing the indicated concentrations of tigecycline, colistin or amikacin.** Replicate experiments gave results similar to those shown.

Among the 68 tested isolates, 85.3% (58 isolates) was susceptible to tigecycline, 97.1% (66 isolates) was susceptible to colistin, and 51.5% (35 isolates) was susceptible to amikacin. Thirty isolates were susceptible to all the three drugs, and they were mainly grouped into four clonal types (A, B, C, D) identified by ERIC-PCR (**Figure 2**). The MPC distributions of the 30 isolates are listed in **Table 1**. The MPC_90_ for tigecycline, colistin, and amikacin were 16, >128, and 128 mg/L, respectively. One representative isolate of each clone type (Kp3 for A, Kp18 for B, Kp8 for C, and Kp23 for D) was randomly selected for evaluating the effect of antimicrobial concentrations on the selection of resistant mutants. As shown in **Figure 3**, a distinct plateau region was observed in the recovery curves of colistin. When the colistin concentration increased from 2- to 16-fold of the MIC, the frequency of resistant mutants did not decrease significantly.

**Table 1:** MPCs distribution of three antimicrobials alone for 30 clinical isolates of KPC-producing *Klebsiella pneumoniae*.

Antimicrobials (μg/ml)	4	8	16	32	64	128	>128	MPC_90_	MPC_90_/MIC_90_
Tigecycline	6	11	12	1				32	8
Colistin					2	4	24	>128	>256
Amikacin				6	20	4		128	32


### MPCs and MSWs of Drug Combinations

**FIGURE 4:**
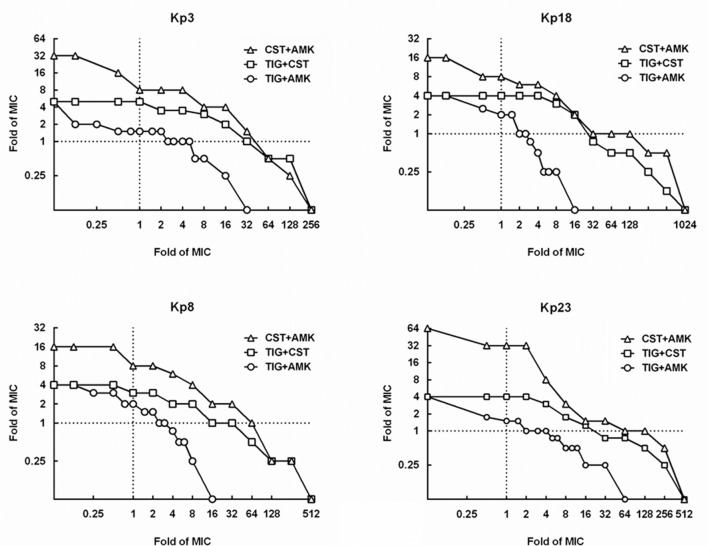
**Mutant selection window of drug combinations (MSW_combination_) for four KPC-producing *K. pneumoniae* strains.** MSW_combination_ can be measured as the area that extends from the MPC curve (curves with symbols) to the MIC lines (dashed line). TIG, tigecycline; CST, colistin; AMK, amikacin.

**FIGURE 5:**
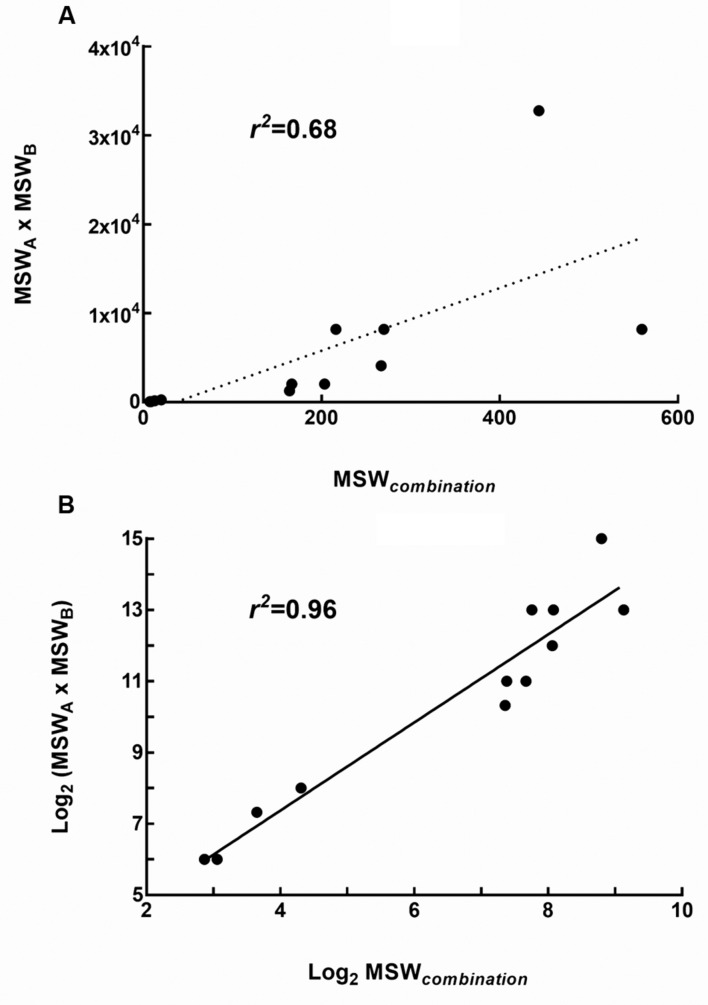
**Relationship between the MSW of single drugs and the MSW of drug combinations (MSW_combination_).** **(A)** Correlation between MSW_combination_ and the product of MSW of each single drug (MSW_A_ × MSW_B_); **(B)** Correlation between log2 MSW_combination_ and log2 (MSW_A_ × MSW_B_).

The MPCs of three pairwise drug combinations were shown in **Figure 4**. The average MSW*_combination_* of tigecycline-amikacin, tigecycline-colistin, and amikacin-colistin combinations for 4 strains were 11.99, 200.13, and 372.38, respectively. The MSW*_combination_* of tigecycline-amikacin combination was much smaller than that of the other two combinations (*P* = 0.002). A correlation was found between the MSW*_combination_* and the product of MSW of each single drug (MSW_A_ × MSW_B_; *r^2^* = 0.68, *P* = 0.0153), and a strong correlation was found between log2 MSW*_combination_* and log2 (MSW_A_ × MSW_B_; *r^2^* = 0.96, *P* < 0.0001; **Figure 5**).

Combinations of 1MIC multiple amikacin and 1.5–2 MIC multiple tigecycline could restrict the recovery of resistant mutants on agar plates, while resistant mutants still recovered on plates containing 1 MIC multiple amikacin and 32 MIC multiple colistin in strain Kp3, Kp8, and Kp23 (**Figure 4**). As displayed in **Table 2**, 2 MIC multiple colistin in combination with 2 MIC multiple tigecycline or amikacin merely resulted in approximately 10-fold decrease in mutational frequency relative to their individual mutational frequencies. By contrast, combinations of 1MIC multiple tigecycline and 1MIC multiple amikacin could result in 1000- to 10000-fold reduction in mutational frequency for all strains.

**Table 2:** Mutational frequencies of drug-isolate combinations at 1 MIC and 2 MIC multiple.

Isolate	MIC multiple	TIG	CST	AMK	TIG+ CST	TIG+ AMK	AMK+CST
Kp3	1×	2.07 × 10^-5^	8.52 × 10^-5^	1.79 × 10^-5^	4.15 × 10^-6^	7.04 × 10^-9^	4.52 × 10^-7^
	2×	2.68 × 10^-6^	7.85 × 10^-6^	2.28 × 10^-6^	2.73 × 10^-7^	—	1.90 × 10^-7^
Kp18	1×	6.95 × 10^-5^	6.75 × 10^-5^	1.65 × 10^-5^	2.95 × 10^-6^	1.54 × 10^-8^	2.85 × 10^-6^
	2×	1.86 × 10^-7^	3.18 × 10^-6^	3.93 × 10^-6^	6.50 × 10^-8^	—	5.02 × 10^-7^
Kp8	1×	6.83 × 10^-5^	5.45 × 10^-5^	2.75 × 10^-5^	4.18 × 10^-6^	6.28 × 10^-8^	4.16 × 10^-6^
	2×	2.17 × 10^-7^	6.45 × 10^-6^	1.02 × 10^-5^	7.23 × 10^-8^	—	1.49 × 10^-6^
Kp23	1×	3.18 × 10^-5^	3.82 × 10^-5^	1.51 × 10^-5^	8.15 × 10^-6^	4.50 × 10^-9^	1.85 × 10^-6^
	2×	8.17 × 10^-7^	4.93 × 10^-6^	9.24 × 10^-7^	4.36 × 10^-8^	—	6.76 × 10^-7^


*TGC, tigecycline; CST, colistin; AMK, amikacin; —, no colony recovered.*

### Characterization of Recovered Resistant Mutants

**FIGURE 6:**
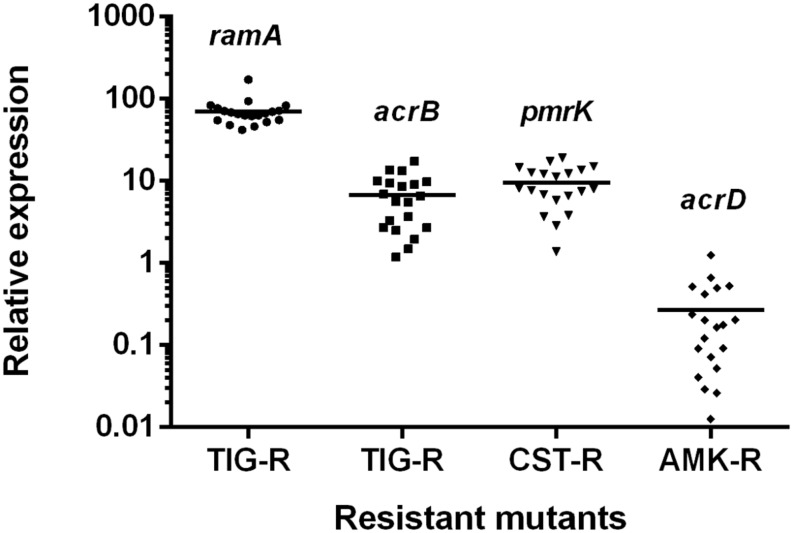
**Relative expression levels of *ramA*, *acrB*, *pmrK*, and *acrD* genes for resistant mutants of Kp3 recovered from the selection pressure of three different drugs.** TIG-R, tigecycline-resistant mutants; CST-R, colistin-resistant mutants; AMK-R, amikacin-resistant mutants.

The possible resistance mechanisms of recovered resistant mutants from the Kp3 isolate were further characterized. As shown in **Figure 6**, the average expression levels of *ramA* and *acrB* genes for 20 tigecycline-resistant mutants (Kp3-T1 to Kp3-T20) increased to 70.4 and 6.8 times compared with the parent isolate. The average expression level of *pmrK* genes for colistin-resistant mutants (Kp3-D1 to Kp3-D20) increased to 9.5 times compared with the parent isolate. But the expression levels of *acrD* genes were down-regulated in most amikacin-resistant mutants. Nine colistin-resistant mutants were detected to carry mutations in *mgrB* (GenBank accession numbers KX650149 for Kp3-D3; KX650150 for Kp3-D4; KX650151 for Kp3-D5 and Kp3-D8; KX650152 for Kp3-D6, Kp3-D13, Kp3-D15, and Kp3-D17; KX650153 for Kp3-D18), whereas PCR results of three mutants were negative. Because all the mutants were derived from the same parent isolate, we deemed that these three colistin-resistant mutants with negative PCR results had mutations in *mgrB* as well. Of the amikacin-resistant mutants, none were detected to harbor the screened aminoglycoside-modifying enzymes genes or 16S rRNA methylase genes.

## Discussion

Although the process of antibiotic-resistance evolution is quite complicated, and resistance may occur when bacteria are exposed to quite low antibiotic concentrations, resistant mutants with strong phenotypes are much more likely selected under the pressure of lethal drug concentrations (approximately from MIC to MPC; Hughes and Andersson, 2012). Therefore, the determination of MPC and MSW of a given antibiotic could be useful in telling whether it is reasonable to use monotherapy with great chance of resistant mutants emerging. Our study showed the MPCs of tigecycline, amikacin and colistin for KPC-producing *K. pneumoniae* were very high. The outcomes are in agreement with recent studies which reported that the MPCs of tigecycline and colistin for *K. pneumoniae* ranged between 4–16 mg/L and >128 mg/L, respectively (Choi and Ko, 2014; Choi et al., 2015). Moreover, we found a broad plateau in the recovery curves of colistin, indicating that highly resistant subpopulations could be easily selected at low drug concentrations. The *in vitro* results might partly explain why the rapid development of resistance to these agents during long-term monotherapy has been frequently reported.

Many studies have demonstrated that combination therapy could narrow or close the MSW, thereby minimizing or preventing the selection of resistant bacterial cells (Zhanel et al., 2006; Cai et al., 2012; Ni et al., 2013). We found just like drug combinations displayed synergistic or antagonistic effects on inhibiting bacterial growth, different combinations also showed different ability to affect the evolution of resistance. The addition of low-dose colistin to tigecycline or amikacin could not decrease the recovery of resistant mutants, while low-dose tigecycline-amikacin combination could significantly reduce the mutational frequency. Lübbert et al. (2013) reported that when both gentamicin and colistin were used for selective digestive decontamination in patients with KPC-producing *K. pneumoniae*, rapid emergence of secondary resistance to gentamicin (45% patients) and colistin (19% patients) were observed. Another two studies also reported that resistant strains were isolated during the combination therapy of colistin and tigecycline (Elemam et al., 2009; Cho et al., 2012). Therefore, the combinations of colistin-tigecycline and colistin-aminoglycosides may not effectively suppress the selection of resistant mutants when treating KPC-producing *K. pneumoniae* infections in clinic. In addition, we found a strong correlation between the MSW*_combination_* and the MSW of individual drugs. This suggests that combinations including individual drug constituents with smaller MSWs may have better ability in preventing the evolution of resistance.

In our study, relatively increased expression levels of *acrB* and *ramA* genes were detected in all tigecycline-resistant mutants. These results are consistent with the notion that up-regulation of efflux pumps is associated with tigecycline resistance in Gram-negative bacteria (Wang et al., 2015). Previous studies found the inactivation or down-regulation of *mgrB* gene is responsible for colistin resistance in *K. pneumoniae* of clinical origin (Cannatelli et al., 2014; Poirel et al., 2015). Among 20 tested colistin-resistant mutants in this study, mutations in *mgrB* genes were also identified in 12 mutants. No aminoglycoside-modifying enzymes genes or 16S rRNA methylase genes were detected in amikacin-resistant mutants. Though the efflux pump AcrD was shown to participate in the efflux of aminoglycosides (Rosenberg et al., 2000), interestingly, the *acrD* gene of resistant mutants were down-regulated in this study. The spontaneous resistance to amikacin might involve other mechanisms, such as inadequate drug transport and alterations of target ribosomal binding sites (Bryan, 1988).

Limitations exists in our study. In clinical conditions, *K. pneumoniae* can acquire resistance via chromosomal mutations and lateral gene transfer (Smith et al., 2003). Our study could not reflect the whole complicated clinical picture, because MPCs/MSWs derived from chromosomal mutations but not lateral gene transfer. Additionally, constant antibiotic concentrations were used throughout this *in vitro* study, and we neglected the influence of *in vivo* immunity on resistance selection. Therefore, studies using ideally animal models are needed to confirm these findings.

## Conclusion

The wide MSWs of tigecycline, colistin, and amikacin for KPC-producing *K. pneumoniae* suggest the possibility of rapid emergence and spread of resistance during long-term monotherapy in medical practice. Compared with the colistin-tigecycline and colistin-amikacin combinations, the tigecycline-amikacin combination can effectively suppress the selection of resistance at low concentrations. Combinations including individual drug constituents with smaller MSWs may have better ability in preventing the evolution of resistance in clinic.

## Author Contributions

WN, CW, CZ, JC, and YL designed and performed the experiments; WN, JZ, BL, and JC analyzed the data, outlined and wrote the manuscript; JC, RW, and YL were involved in data discussion and corrected the manuscript. All authors reviewed the manuscript.

## Conflict of Interest Statement

The authors declare that the research was conducted in the absence of any commercial or financial relationships that could be construed as a potential conflict of interest.
