# Supplementary material for: Tigecycline-Amikacin Combination Effectively Suppresses the Selection of Resistance in Clinical Isolates of KPC-Producing Klebsiella pneumoniae
Source: Front Microbiol. 2016 Aug 19;7:1304. doi: 10.3389/fmicb.2016.01304 (PMC4990548; doi:10.3389/fmicb.2016.01304)
Supplement: Supplementary file 1 [file Table_1.PDF]

Supplementary TABLE 1. Primer sequences used for this study.

Primer	Product	Sequence (5’–3’)	Usage
ERIC-F	ERIC	ATGTAAGCTCCTGGG GATTCAC	ERIC-PCR
ERIC-R		AAGTAAGTGACTGGGGTGAGCG	
acrB-qPCR-F	*acrB*	AAACTTCGCCACTACGTCATA	qRT-PCR
acrB-qPCR-R		AGCTTAACGCCTCGATCAT	
ramA-qPCR-F	*ramA*	GATATCGCTCGCCATGC	qRT-PCR
ramA-qPCR-R		CTGTGGTTCTCTTTGCGGTAG	
acrD- qPCR-F	*acrD*	CCTTTGTCAAAGCCTCGATTATC	qRT-PCR
acrD- qPCR-R		CAGGAACAGATACATCACCAG	
pmrK- qPCR-F	*pmrK*	CGCTGAATATGCTCGACCCAGAAG	qRT-PCR
pmrK- qPCR-R		GCTGGCGGTAATCGTCTGTACG	
rrsE-qPCR-F	*rrsE*	GTCATCATGGCCCTTACGAG	qRT-PCR
rrsE-qPCR-R		ACTTTATGAGGTCCGCTTGCT	
mgrB-F	*mgrB*	CACCACCTCAAAGAGAAGGCGTTC	PCR and sequencing
mgrB-R		AACACGTTTTGAAACAAGTCGATGATTC	
aac (3)-I-F	*aac (3)-I*	ACCTACTCCCAACATCAGCC	PCR
aac (3)-I-R		ATATAGATCTCACTACGCGC	
aac (3)-II-F	*aac (3)-II*	ACTGTGATGGGATACGCGTC	PCR
aac (3)-II-R		CTCCGTCAGCGTTTCAGCTA	
aac (3)-III-F	*aac (3)-III*	CACAAGAACGTGGTCCGCTA	PCR
aac (3)-III-R		AACAGGTAAGCATCCGCATC	
aac (6’)-I-F	*aac (6’)-I*	TATGAGTGGCTAAATCGA	PCR
aac (6’)-I-R		CCCGCTTTCTCGTAGCA	
aac (6’)-II-F	*aac (6’)-II*	TTCATGTCCGCGAGCACCCC	PCR
aac (6’)-II-R		GACTCTTCCGCCATCGCTCT	
ant (2”)-I-F	*ant (2”)-I*	GAGCGAAATCTGCCGCTCTGG	PCR
ant (2”)-I-R		CTGTTACAACGGACTGGCCGC	

ant (3")-I-F<br>*ant (3")-I*-R	*ant (3")-I*	TGATTTGCTGGTTACGGTGAC<br>CGCTATGTTCTCTTGCTTTTG	PCR
aph(3')-VI-F<br>*aph(3')-VI*-R	*aph(3')-VI*	ATACAGAGACCACCACCATACAGT<br>GGACAATCAATAATAGCAAT	PCR
armA-F<br>*armA*-R	*armA*	TATGGGGGTCTTACTATTCTGCCTAT<br>TCTTCCATTCCCTTCTCCTTT	PCR
rmtA-F<br>*rmtA*-R	*rmtA*	CTAGCGTCCATCCTTTCCTC<br>TTTGCTTCCATGCCCTTGCC	PCR
rmtB-F<br>*rmtB*-R	*rmtB*	TCAACGATGCCCTCACCTC<br>GCAGGGCAAAGGTAAAATCC	PCR
rmtC-F<br>*rmtC*-R	*rmtC*	GCCAAAGTACTCACAAGTGG<br>CTCAGATCTGACCCAACAAG	PCR
rmtD-F<br>*rmtD*-R	*rmtD*	CTGTTTGAAGCCAGCGGAACGC<br>GCGCCTCCATCCATTCGGAATAG	PCR
npmA-F<br>*npmA*-R	*npmA*	CTCAAAGGAACAAAGACGG<br>GAAACATGGCCAGAAACTC	PCR
